# In-Silico Modeling to Compare Radiofrequency-Induced Thermal Lesions Created on Myocardium and Thigh Muscle

**DOI:** 10.3390/bioengineering9070329

**Published:** 2022-07-19

**Authors:** Juan J. Pérez, Enrique Berjano, Ana González-Suárez

**Affiliations:** 1 BioMIT, Department of Electronic Engineering, Universitat Politècnica de València, 46022 Valencia, Spain; jjperez@eln.upv.es (J.J.P.); eberjano@eln.upv.es (E.B.); 2 Electrical and Electronic Engineering, Translational Medical Device Lab, National University of Ireland Galway, H91 TK33 Galway, Ireland

**Keywords:** beating heart, cardiac ablation, computer modeling, thigh muscle, radiofrequency ablation

## Abstract

Beating heart (BH) and thigh muscle (TM) are two pre-clinical models aimed at studying the lesion sizes created by radiofrequency (RF) catheters in cardiac ablation. Previous experimental results have shown that thermal lesions created in the TM are slightly bigger than in the BH. Our objective was to use in-silico modeling to elucidate some of the causes of this difference. In-silico RF ablation models were created using the Arrhenius function to estimate lesion size under different energy settings (25 W/20 s, 50 W/6 s and 90 W/4 s) and parallel, 45° and perpendicular catheter positions. The models consisted of homogeneous tissue: myocardium in the BH model and striated muscle in the TM model. The computer results showed that the lesion sizes were generally bigger in the TM model and the differences depended on the energy setting, with hardly any differences at 90 W/4 s but with differences of 1 mm in depth and 1.5 m in width at 25 W/20 s. The higher electrical conductivity of striated muscle (0.446 S/m) than that of the myocardium (0.281 S/m) is possibly one of the causes of the higher percentage of RF energy delivered to the tissue in the TM model, with differences between models of 2–5% at 90 W/4 s, ~9% at 50 W/6 s and ~10% at 25 W/20 s. Proximity to the air–blood interface (just 2 cm from the tissue surface) artificially created in the TM model to emulate the cardiac cavity had little effect on lesion size. In conclusion, the TM-based experimental model creates fairly similar-sized lesions to the BH model, especially in high-power short-duration ablations (50 W/6 s and 90 W/4 s). Our computer results suggest that the higher electrical conductivity of striated muscle could be one of the causes of the slightly larger lesions in the TM model.

## 1. Introduction

Radiofrequency (RF) cardiac ablation (RFCA) is a catheter-based procedure broadly used to cure cardiac arrhythmias. Many innovations have recently been introduced in RF catheters to achieve safer and more effective RF-induced lesions. Regulatory agencies require clinical evaluations based on clinical and preclinical data before the approval and subsequent commercialization of a new RF catheter. Preclinical data are basically obtained from bench testing, which can be based on different models, such as in-silico (computer-based), agar-phantom, ex vivo and in vivo models; in vivo models being most similar to clinical scenarios. To date, two in vivo models have been proposed to assess the lesions created by different RF catheters: beating heart (BH) and thigh muscle (TM). While the former consists of applying the RF catheter directly to the myocardium (as in RFCA in clinical practice), the latter uses the striated muscle of an animal’s thigh as the target to create RF-induced lesions. This second model consists of making an incision on the thigh muscle, raising the skin and connective tissue to create a cradle overlaying the muscle, filling the cradle with the animal’s heparinized blood at 37–38 °C, and recirculating it to mimic the effect of the circulating blood in the cardiac chamber [1]. To our knowledge, only the study by Leshem et al. [2] compared the thermal lesions created by the TM and BH models. They found that at low energy settings (30 W for 40 s), the TM model created wider lesions (12.2 mm vs. 9 mm) whereas lesion depths were similar (5.7–6.0 mm). At higher energy settings (40 W for 60 s), lesions were also larger in the TM model, with differences of ~0.7 mm in depth and ~5.0 mm in width [2]. The authors concluded that TM preparation is a reasonable model for evaluating RF catheter technologies, even though it may overestimate lesion size. This overestimation was also found in other recent studies. For instance, the same group reported similar depths and ~2 mm wider lesions with the TM model using a standard catheter at 40 W/60 s [3], while Nakawaga et al. [4] reported slightly larger lesions (~15%) with the TM model at different energy settings (90 W/4 s, 50 W/10 s, 30 W/30 s).

Leshem et al. [2] suggested several reasons for the difference in lesion sizes between the TM and BH models, such as changes in the electrode–tissue contact surface (induced by cardiac movements or the irregular cardiac surface), in addition to the different tissue properties of striated muscle and myocardium. In-silico modeling can study the weight of each factor since it isolates and controls each one. Our objective was thus to use an in-silico model to determine the reasons why the TM model creates slightly larger lesions than the BH model. The effect of the catheter position and thermal anisotropy was assessed. We also considered an ‘intermediate’ model based on the TM model geometry but with the same properties as the myocardium in order to assess the effect of the possible alteration of the RF current distribution around the electrode in the TM model, since the current cannot pass through the blood surface due to the presence of air.

## 2. Methods

### 2.1. Model Geometry

**Figure 1:**
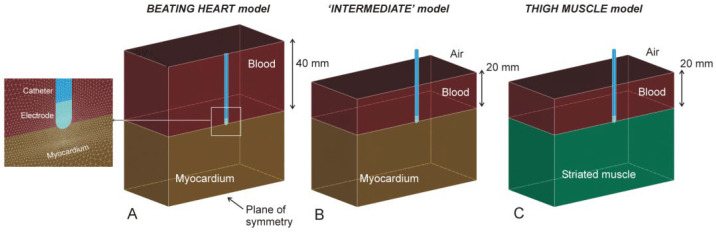
Geometries of models of myocardium (**A**), thigh muscle (**C**) and ‘intermediate’ (**B**) (see text for details). The figures are those of the case of a perpendicular catheter.

Three-dimensional computational models were built including a 7.5 Fr–3.5 mm irrigated electrode placed on the tissue and surrounded by blood. Two tissue types were considered: myocardium for the BH and striated muscle for the TM model. Perpendicular (90°), 45° and horizontal (0°) positions between the tissue surface and the catheter axis were also considered. The perpendicular electrode was assumed to be inserted 0.5 mm, which is a low contact force [5]. In the other positions, the electrode was inserted to obtain the same electrode–tissue contact surface as in the perpendicular case (0.193 mm for horizontal and 0.498 mm for 45°). Due to there being only one symmetry plane, the model only considered half the real volume. Figure 1 shows the geometries of the different models considered. Note that all the models were really limited-domain, i.e., with only a representative volume of tissue around the RF catheter, so that the position of the dispersive patch was assumed as an electrical boundary condition (0 V) set on some of the outer limits. The BH model (Figure 1A) consisted of a homogeneous fragment of myocardium, blood around the catheter and a boundary condition of 0 V set on all the outer limits (as described in [5,6]). Note that although the BH model is actually based on mechanically contracting tissue, previous results suggested that heartbeat-induced catheter motion has little impact on lesion size [7], indicating that the BH model was assumed to be composed of non-contracting tissue.

The TM model (Figure 1C) consisted of a homogeneous fragment of striated muscle, blood around the catheter up to a height of 20 mm (as described in the experimental setup of the thigh model [1]) and a boundary condition of 0 V set on all the outer limits, except on the upper surface, since it is the air-blood interface. We also considered an ‘intermediate’ model (Figure 1B), with the same geometry as the TM model but with the properties of the myocardium. This model was intended to assess the effect of the possible alteration of the RF current distribution around the electrode in the TM model since the current cannot pass through the blood surface due to the presence of air.

Each model (half of the entire domain) was made up of ~214,000 tetrahedral elements and ~300,000 nodes. The outer dimensions (4 cm around the ablation electrode), mesh size (minimum of 118 μm around the electrode and maximum of 12 mm in the periphery) and the time step (ranging from 20 to 100 ms) were verified by means of a convergence test, as described in [7], i.e., using lesion depth and maximum temperature as convergence parameters, and 0.5 mm and 1 °C as convergence criteria.

### 2.2. Power-Duration Settings

Computer simulations were conducted to mimic three power-duration settings used in RFCA: moderate power–moderate duration (MPMD, 25 W for 20 s), high power–short duration (HPSD, 50 W for 6 s) and very high power–very short duration (vHPvSD, 90 W for 4 s). The power value used in the simulations was reduced by 20% since the model did not include the entire torso [6]. The simulation data were obtained for up to 90 s after RF onset to determine the lesion growth due to thermal latency.

### 2.3. Tissue Properties

Tissue properties were taken from the IT’IS Foundation database [8], while the ablation catheter properties were taken from Pérez et al. [9] (see Table 1). Blood thermal properties were not included since the thermal problem was not solved in that sub-domain. Tissue electrical conductivity was assumed to change with temperature by 1.5%/°C and drop drastically two orders of magnitude between 99 and 101 °C to simulate dehydration by vaporization. 

Previous experimental studies have suggested that both the myocardium and striated muscle barely show electrical anisotropy at 500 kHz [10,11], while certain thermal anisotropy has been experimentally observed in muscle, for instance by Yue et al. [12], with a value ~30% higher in the longitudinal than in the transverse orientation. With this value in mind, we conducted an additional set of simulations with the BH model to assess the impact of possible thermal anisotropy on lesion size. For this, we considered two different values of *k*, which differed by 30% and whose mean value was identical to that considered in the isotropic model (i.e., 0.56 W/m·K, see Table 1): 0.487 W/m·K for transverse (i.e., perpendicular to the tissue surface) and 0.633 W/m·K for longitudinal orientation (i.e., parallel to the tissue surface). This implied assuming that the orientation of the fibers is mainly parallel to the surface of the tissue, as occurs mainly in the myocardium (the largest area of the heart wall comprised of muscle tissue) and to a lesser extent in the endocardium and epicardium [13].

### 2.4. Governing Equations

The model solved a coupled electric–thermal problem numerically using the Finite Element Method on ANSYS software (ANSYS, Canonsburg, PA, USA). The governing equation for the thermal problem was the Bioheat Equation [14]:

(1) $$\rho c\frac{\partial T}{\partial t}=\nabla \left(k\nabla T\right)+Q_{RF}+Q_{P}+Q_{m}$$

where *ρ* is density (kg/m^3^), *c* specific heat (J/kg·K), *T* temperature (°C), *t* time (s), *k* thermal conductivity (W/m·K), *Q_RF_* the heat source caused by RF power (W/m^3^), *Q_p_* the heat loss caused by blood perfusion (W/m^3^) and *Q_m_* the metabolic heat generation (W/m^3^). Both *Q_m_* and *Q_p_* were ignored as these terms are negligible compared to the others [14]. 

A quasi-static approximation was employed for the electrical problem. The electrical field ***E*** distribution was obtained from ***E*** *=* −∇*Φ*, *Φ* being voltage, which was obtained from ∇·(*σ*(*Τ*)∇*Φ*) = *0*, *σ* being electrical conductivity. The heat source caused by RF power *Q_RF_* was then obtained as *Q_RF_ = σ* |***E***|^2^.

In order to model the vaporization in the tissue, Equation (1) was written as a balance of enthalpy changes instead of the energy changes proposed in [15]:

(2) $$\frac{\partial h_{t}}{\partial t}=\nabla \left(k\nabla T\right)+Q_{RF}\;$$

where *h_t_* is the tissue enthalpy per unit volume. This value can be determined by assessing the amount of energy deposited in the tissue when its temperature is raised from 37 °C to values above 100 °C. According to [15], enthalpy per unit volume is:

(3) $$h_{t}=\{\begin{array}{c} \rho_{h}c_{h}\left(T-37\right),\;37\leq T\leq 99\;{}^{\circ}C\;\\ \rho_{h}c_{h}\left(99-37\right)+H_{t}\cdot \frac{\left(T-99\right)}{\left(100-99\right)}\;,\;99<T\leq 100\;{}^{\circ}C\;\\ \rho_{h}c_{h}\left(99-37\right)+H_{t}+\rho_{dh}c_{dh}\left(T-100\right),\;T>100\;{}^{\circ}C \end{array}$$

where the subscript *h* refers to the properties of the hydrated tissue (i.e., before reaching 99 °C), the subscript *dh* refers to those of the dehydrated tissue and *H_t_* is the tissue vaporization latent heat. The partial derivative of the enthalpy in Equation (1) can be therefore expressed as:

(4) $$\frac{\partial h_{t}}{\partial t}=\{\begin{array}{c} \rho_{h}c_{h}\frac{\partial T}{\partial t},\;37\leq T\leq 99\;{}^{\circ}C\\ \frac{H_{t}}{∆T}\cdot \frac{\partial T}{\partial t}\;,\;99<T\leq 100\;{}^{\circ}C\;\\ \rho_{dh}c_{dh}\frac{\partial T}{\partial t},\;T>100\;{}^{\circ}C \end{array}$$

where ∆*T* = 1 °C. The parameters used to model the phase change (vaporization) were as follows: *H_t_* was estimated as the product of the water vaporization latent heat (*H_w_*) and the water mass fraction in the tissue (*C*). *H_w_* was calculated as the product of the water vaporization latent heat (2256 kJ/kg) and water density (958 kg/m^3^), both assessed at 100 °C (www.engineeringtoolbox.com/water-properties-d_1573.html; accessed 19 July 2022), given a value of 2.161 × 10^9^ J/m^3^. *C* was considered to be 73.7% in the myocardium and 79.5% in striated muscle [16]. Values of density and specific heat for dehydrated tissue were assumed to be identical to hydrated tissue since a previous study showed that the impact of modeling the thermal properties of dehydrated tissue is minimal (differences in lesion size below 0.03 mm [7]). The described governing equations were the same in the cases of myocardium and striated muscle.

### 2.5. Boundary Conditions

The electrical boundary conditions were as follows: the dispersive electrode was simulated using conditions of 0 V on all the outer surfaces, except the symmetry plane and the upper surface in the TM and ‘intermediate’ model, in which a condition of zero electrical current was set (Figure 1B,C). Zero current was applied in the plane of symmetry. The electrical voltage in the ablation electrode was modulated to keep the total power applied constant throughout the RF pulse.

The thermal boundary conditions were the following: the circulating blood produces convective heat transfer, which can be modeled using Newton’s Law of cooling in which the convective heat transfer rate is

(5) $$Q_{conv}\;=\;h\;(T\;-\;T_{B})$$

where *h* is the convective heat transfer coefficient, *T_B_* is the blood temperature (37 °C) and *T* is the surface temperature of the tissue. A thermal boundary condition based on Equation (5) was set on the electrode–blood and myocardium–blood interfaces, with thermal transfer coefficients of 3310 W/m^2^·K and 694 W/m^2^·K, respectively. These values corresponded with a flow velocity of 0.1 m/s, simulating ablation sites with low local blood flow, such as in patients with chronic atrial fibrillation and dilated atria [4]. A temperature of 37 °C was set at the outer tissue contours to simulate the body temperature. The initial temperature was also 37 °C. Electrode irrigation was modeled by fixing a value of 25 °C in the cylindrical zone of the electrode tip and leaving the semispherical tip free, which mimics a multi-hole electrode, since we assumed that irrigation occupied almost the entire surface of the electrode [17]. 

### 2.6. Analyzed Outcomes

Lesion size, quantified by depth (D) and maximum width (MW), was computed from the thermal damage index Ω based on the Arrhenius model, which relates the number of undamaged cells *C*(0) present before heating to the remaining number of undamaged cells at time *t* indicated by *C(t)* as follows:

(6) $$\Omega (t)=\ln(\frac{C(0)}{C(t)})$$

This can be computed from the ‘thermal history’ to which the tissue is subjected, specifically from temperature *T* (in Kelvin) reached at different times *t* (s) of heating:

(7) $$\Omega (t,T)=\int_{0}^{t}Ae^{\left[-E_{a}/RT(\tau )\right]}d\tau$$

where *A* is the frequency factor, *E_a_* is the activation energy and *R* is the universal gas constant (8.3143 J/mol·K). Although the values of *A* and *E_a_* are different for each tissue type and analyzed process, we considered *A* = 7.39 × 10^39^ s^−1^ and *E_a_* = 2.577 × 10^5^ J/mol as was the case in [18]. These values have been widely used in computer modeling of RF ablation processes, even though they do not specifically refer to cardiac tissue. We previously checked that they were able to provide a Ω = 1 isoline more or less coincident with the 72 °C isotherm for 5 s heating [19] and more or less coincident with the 55 °C isotherm after 60 s of heating [20,21]. The Ω = 1 isoline was therefore considered to represent the thermal lesion contour, which is equivalent to a cell death probability of 63% [14].

We computed the percentage of power targeted on the tissue (P_T_), i.e., myocardium in the BH model and striated muscle in the TM model. The remaining percentage was assumed to be ‘lost’ in the blood and so did not contribute to the RF-induced heating. P_T_ values were computed at the beginning of each RF pulse. The percentage of total energy targeted on the tissue at the end of each RF pulse (E_T_) was also computed. This was based on the volumetric density of electrical energy deposited in each element of the model (in W/m^3^) and integrating each subdomain (tissue and blood).

## 3. Results

### 3.1. Comparison of Lesion Sizes

Table 2 shows the lesion size computed with the BH model for the different power–duration settings and catheter position at the end of the RF pulse and 90 s after the pulse onset. In all the catheter positions the lesions at 25 W/20 s were slightly deeper and narrower than at 90 W/4 s, which indicated increasing width/depth ratios from 1.7–1.8 to ~2.0 as the pulse shortens and power increases. The lesion size computed by the ‘intermediate’ model was almost identical to the BH model, with <0.05 mm differences in depth, <0.3 mm in width and <2.1 mm^3^ in volume.

**Figure 2:**
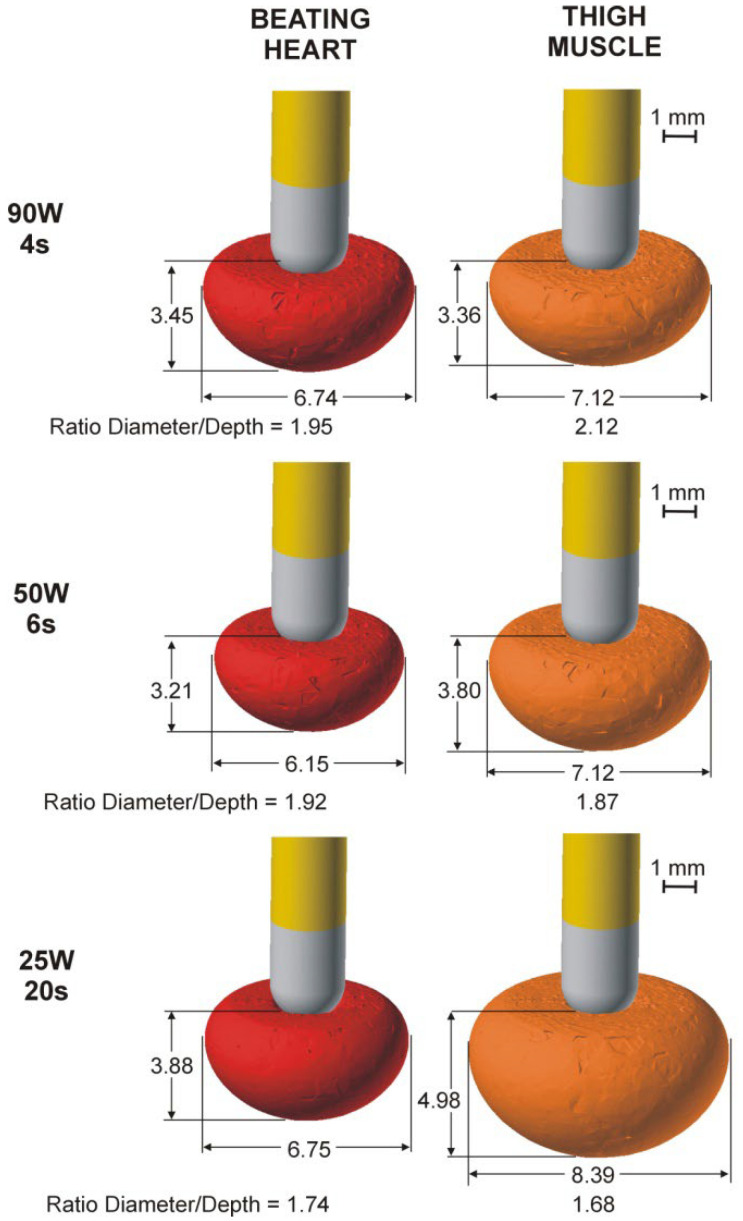
Lesion geometries (computed with Ω = 1 isoline after 90 s from the Arrhenius model) in the case of the perpendicular catheter for both models (beating heart and thigh muscle) and the three power settings (90 W/4 s, 50 W/6 s and 25 W/20 s).

Table 3 shows the lesion size computed with the TM model. Regardless of catheter position, lesions were bigger at 25 W/20 s than 90 W/4 s and 50 W/6 s, and generally larger than in the BH model. Interestingly, the differences between the TM and BT models increased with ablation time; lesion depths were almost identical at 90 W/4 s (differences less than 0.1 mm), but were 0.6–0.7 mm deeper at 50 W/6 s and ~1.1 mm deeper in the TM model at 25 W/20 s. Likewise, the TM and BT model lesion widths were almost identical at 90 W/4 s (differences less than 0.5 mm) but were ~1 mm wider in the TM model at 50 W/6 s and 1.5–1.6 mm wider at 25 W/20 s. Figure 2 shows lesion shapes computed for the BH and TM models and power settings with perpendicular catheters. 

Including thermal anisotropy in the BT model had little impact on lesion size, with differences of less than 0.1 mm in depth and 6.5 mm^3^ in volume between the isotropic and anisotropic cases. The lesion was slightly wider in the direction of fiber orientation (0.2–0.3 mm) than the isotropic case, but almost identical in the transverse direction (differences less than 0.1 mm).

### 3.2. Electrical Performance

Table 4 shows the percentages of power (P_T_) and energy (E_T_) targeted on the tissue for both models. The P_T_ and E_T_ values in the ‘intermediate’ model differ only by ~1% with respect to the BH model. In contrast, the TM model showed P_T_ and E_T_ values significantly greater than the BH model (24.4 ± 0.8% vs. 16.8 ± 0.6% and 27.3 ± 3.4% vs. 19.8 ± 0.8%, respectively). While the difference in P_T_ between both models was relatively independent of the power setting (~7.6 ± 0.2%), the difference in E_T_ depended on the power setting, with increasing values as power decreased and pulse duration extended (2–5% for 90 W/4 s, ~9% for 50 W/6 s and ~10% for 25 W/20 s).

### 3.3. Effect of Blood Quantity on the Thigh Muscle

**Figure 3:**
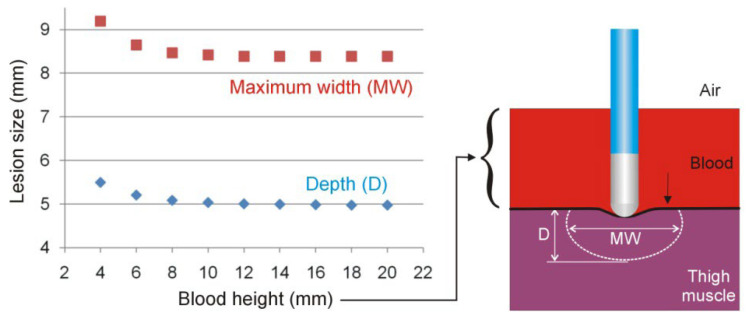
Lesion sizes (depth and maximum width) for different blood heights in the thigh model in the case of 25 W/20 s and perpendicular catheter orientation.

Figure 3 shows the effect of the height of the heparinized blood on the lesion size for 25 W/20 s and perpendicular catheter orientation. As observed, the lesion size hardly changes when the blood height is greater than 10 mm.

## 4. Discussion

### 4.1. Main Findings

An RFCA in-silico model was developed to explore the differences in lesion size created in beating heart and thigh muscle models. Both models considered the electrical and thermal properties of the myocardium or striated muscle to represent the biophysical phenomena involved. The key findings were as follows:

(1) The beating heart and ‘intermediate’ models predict nearly identical lesions, suggesting that the blood pool artificially created on the thigh to emulate the cardiac cavity has little effect on lesion size. In other words, proximity to the air–blood interface (just 2 cm from the tissue surface) does not appear to affect the distribution of RF current. Computer results suggest that a minimum height of 10 mm for the blood pool must be included in the thigh model.
(2) The different electrical conductivities of striated muscle (in the thigh model) and myocardium (beating heart model) might be responsible for the slight difference in lesion size between models.
(3) The difference in lesion sizes between the thigh and beating heart models seems to depend on the energy setting, with hardly any differences at 90 W/4 s, but with differences of 1 mm in depth and 1.5 m in width at 25 W/20 s.
(4) The fact that more energy is delivered to the tissue in the thigh model, with differences that increase with RF pulse duration, is possibly the cause of the differences in the different lesion sizes of the models.

Our study aimed to compare the lesion sizes in two experimental models used in pre-clinical research on cardiac ablation RF catheters: the beating heart model and the thigh muscle model. The reason was to elucidate some of the factors responsible for the reported differences in lesion size between both models [2,3,4]. Although there have been few prior experimental studies on the differences between the RF lesions created by a beating heart and a thigh muscle model, the existing literature suggests that they are a bit larger in the case of the thigh muscle. For example, Leshem et al. [2] reported lesions ~3 mm wider at 30 W/40 s and ~5 mm wider at 40 W/60 s. Lesion depths were similar at 30 W/40 s and 1 mm greater at 40 W/60 s. In a later study, the same authors [3] found hardly any differences in depth and only 2 mm greater width in the thigh model. Overall, our computer results agree with the findings on the slightly larger RF lesions in the muscle model. Interestingly, our results also confirm the trend observed by Leshem et al. [2] regarding the increasing differences in lesion size with the duration of the RF pulse and the distributed energy: from 30 W/40 s (1200 J) to 40 W/60 s (2400 J) in the experimental study by Leshem et al. [2], and from 90 W/4 s (360 J) to 25 W/20 s (500 J) in the present study. We also found that the difference in lesion size between both models might be almost negligible for high-power short-duration ablations (i.e., 50 W/6 s and 90 W/4 s).

Our results suggest that the differences in the thermal and electrical tissue properties involved could have a significant impact on the different lesion sizes of both models. The lesions are larger because the electrical conductivity of striated muscle is greater than that of myocardium at ablation frequencies (0.446 S/m vs. 0.281 S/m), which means more power is targeted on the tissue and therefore less is lost in the blood pool (differences of ~7%). The difference in thermal conductivity (slightly greater in the myocardium) probably has a much smaller impact than electrical conductivity, which determines the power dissipated. As the tissue heats up during the RF pulse, it becomes more conductive (increasing electrical conductivity), allowing the percentage of power targeted on the tissue to increase even more. This is possibly the cause of a higher percentage of final energy distributed in the tissue with longer pulses (20 s vs. 4 s) and greater power (500 J vs. 360 J), causing greater differences in lesion size between both models. In addition, the lower weight of the change in thermal conductivity compared to the change in electrical conductivity was already observed in a previous simulation study using 25 W-30 s [7]. Specifically, a sensitivity analysis was conducted using myocardium to assess the impact of dispersed tissue characteristics on lesion size, considering the maximum and minimum myocardium values reported in the ITIS database (0.5 and 0.6 W/m·K for thermal conductivity, 1059 and 1143 kg/m^3^ for density, 3614 and 3724 J/kg·K for specific heat). The myocardium electrical conductivity was considered to have a ±10% variation, which is of the same order as the dispersions of the rest of the characteristics. The results showed that when electrical conductivity changed ±10%, lesion depth varied between −2.8% and +1%, and maximum width varied between −3.4% and +2.3%. In contrast, the impact of the variation in thermal conductivity was much smaller: when it changed from −11% to +7%, lesion depth barely changed ±0.5%, and maximum width changed ±0.85%.

Our findings suggest that the lesions created on the thigh muscle are slightly greater than those on the myocardium due to striated muscle having higher electrical conductivity. Leshem et al. [2] suggest additional factors, such as changes in the electrode–tissue contact surface induced by heart movements. In this regard, our previous computer modeling results suggest that these movements (at least when they are perpendicular to the myocardial surface) hardly affect lesion size for relatively long RF pulses (30 s) [7]. In fact, in the case of horizontal movement, the electrode slides sideways along the surface, resulting in wider and shallower lesions [22]. It, therefore, does not seem that heartbeat-induced movements of the cardiac wall are a determining factor in the different lesion sizes in both models.

Leshem et al. [2] also suggested the irregular cardiac surface to be the cause of different lesion sizes in the models. They argue that the catheter position on the heart is influenced by curves and trabeculations that can reduce the catheter–tissue interface surface area. We think that these curves and trabeculations could also increase the contact area between the electrode and tissue, increasing the power targeted on the tissue and causing even greater lesions, as Bourier et al. found [23]. Leshem et al. [2] also suggested the difference might lie in the internal blood supply of both tissues. The myocardium is known to be much more perfused than striated muscle (1026 ± 307 vs. 37 ± 13 mL/min·kg [7]). In a previous simulation study, we found that the effect of capillary blood flow on lesion depth increases with the RF pulse. Lesions were deeper in the case of no blood supply (0 mL/min·kg) than 1026 ± 307 mL/min·kg, specifically 0.3 mm for a 30 s RF pulse and 0.7 mm for a 60 s RF pulse [24]. This suggests that the internal blood flow could be partially responsible for the different lesion sizes between the TM and BH models, at least for longer RF pulses.

Compared to the experimental data reported by Leshem et al. [2,3] at 30 W/40 s and 40 W/60 s, we found greater differences in depth between the BH and TM models (~1.1 mm vs. 0.3–0.7 mm), together with a smaller difference in width (1.5–1.6 mm vs. 2–5 mm). In contrast, our results fit perfectly with those found experimentally by Nakawaga et al. [4] at 90 W/4 s, 50 W/10 s and 30 W/30 s (more similar conditions to those modeled by us): they reported that lesion depth and diameter on thigh muscle was ~15% greater than on the beating heart, while we found a difference of 15% in depth (4.12 vs. 3.57 mm) and 14% in width (7.76 vs. 6.80 mm).

Finally, the results including the thermal anisotropy hardly showed any differences in size with respect to the isotropic model, regardless of the power setting, which suggests that this factor has an insignificant impact. Although the recent study by Gu et al. [25] assessed the effect of electrical anisotropy on lesion size after RFCA, the literature does not suggest that electrical anisotropy is important in the myocardium and muscle [10,11].

### 4.2. Practical Implications

The TM model has become the best bench test for RF catheters in cardiac ablation, since it has significant procedural advantages over the BH model, such as better control of catheter position and electrode–tissue contact [2]. However, the experimental data suggests that the TM model slightly overestimates lesion size. The reliability of the TM model must be accompanied by an understanding of the biophysical reasons for the discrepancy in lesion sizes compared to the BH model. Our theoretical/computational analysis suggests two possible causes: (1) the greater electrical conductivity of striated muscle, which allows more power to be targeted on the tissue in the TM model, and (2) the smaller internal blood flow of striated muscle, which reduces the heat-sink effect, favoring the larger lesions in the TM model. Both causes seem to have a greater impact with longer RF pulses, suggesting that there could be little difference between TM and BH with very short RF pulses such as those used with 50 W/6 s and 90 W/4 s. In particular, our results suggest that lesion sizes created with TM and BH are almost identical at 90 W/4 s, with differences less than 0.1 mm in depth and 0.5 mm in width. This has important implications, since recent clinical studies suggest that very high power–very short duration 90 W/4 s radiofrequency (RF) ablation is safe and effective for the treatment of atrial fibrillation [26,27,28,29].

### 4.3. Limitations

First, the mechanical deformation of the tissue surface was not considered, which could have altered the differences in lesion sizes between the models. Note that while we considered the same electrode–tissue contact surface for all the models, differences in their mechanical properties could mean different contact surfaces, and therefore lesion sizes, even with equal contact force. For instance, if we assumed that the myocardium is stiffer than striated muscle, as suggested by Mathur et al. [30] (Young’s modulus of ~100 kPa for cardiac cells vs. ~25 kPa for skeletal muscle cells), then the electrode would go deeper into striated muscle, increasing the contact area, the power targeted on the tissue and consequently, lesion size. This could also contribute to the slightly greater lesion sizes in the thigh muscle. Second, the Arrhenius model considered the same values of *A* and *E_a_* for both BH and TM models. We recognize that the values of these parameters could be different in the myocardium and striated muscle, which could have an impact on assessing lesion size. Third, the mathematical framework used in the current study did not include the dynamics of the steam created when tissue temperature reaches 100 °C, since there is currently no validated model for this purpose. This implies that no conclusion can be drawn from our model about the incidence of steam pops. Despite this limitation, the inclusion of the sudden drop in electrical conductivity around 100 °C as well as the latent heat associated with the phase change, have been shown to be adequate for predicting the lesion sizes in RFCA modeling [14]. Fourth, the values of electrical conductivity at 500 kHz were taken from the ‘Dielectric properties’ section of the IT’IS Foundation database [7] (0.281 S/m for myocardium and 0.446 S/m for striated muscle). It has been pointed out that this database has serious limitations, especially at frequencies below 1 MHz, where the literature values are scarce and have larger than average uncertainties [31]. Compared to these values, other in vivo studies reported more similar values between myocardium (0.54 S/m [10], 0.6 S/m [31]) and striated muscle (~0.6 S/m [11], 0.3–0.5 S/m [31]), which would result in a more similar electrical behavior in both models.

## 5. Conclusions

Our computer results agree with the experimental findings that the lesions in the TM are slightly larger than those in the BH. However, these differences do not seem to be relevant from a clinical point of view, suggesting therefore that the experimental model based on thigh muscle creates reasonably similar-sized lesions to the beating heart model and that the similarity might be even greater for high-power short-duration ablations. The blood pool artificially created on the thigh to simulate cardiac cavity does not appear to affect the distribution of RF current. The differences in electrical conductivity between striated muscle and myocardium might be partially responsible for the differences in lesion size between both models.

## Figures and Tables

**Table 1:** Physical characteristics of tissues and materials of each element used in the models.

Element/Material	*σ* (S/m)	*k* (W/m·K)	*ρ* (kg/m^3^)	*c* (J/kg·K)	Reference
Electrode/Pt-Ir	4.6 × 10^6^	71	21,500	132	[9]
Catheter/Polyurethane	10^−5^	23	1440	1050	[9]
Cardiac Chamber/Blood	0.748	--	--	--	[8]
Striated muscle	0.446	0.49	1090	3421	[8]
Myocardium	0.281	0.56	1081	3686	[8]

*σ*, electrical conductivity (at 500 kHz); *k*, thermal conductivity; *ρ*, density; and *c*, specific heat (all assessed at 37 °C in case of tissue and blood).

**Table 2:** Lesion sizes computed for different power–duration settings and catheter orientation just after the RF pulse (t_RF_) and at 90 s after the onset of the RF pulse (t_90_) in the beating heart model.

		Maximum Depth D (mm)		Maximum Width MW (mm)		Ratio MW/D		Volume (mm^3^)	
Energy Setting	Catheter Orientation	t_RF_	t_90s_	t_RF_	t_90s_	t_RF_	t_90s_	t_RF_	t_90s_
90 W 4 s	0°	2.73	3.54	6.62	7.14	2.42	2.02	70.92	107.46
	45°	2.81	3.54	6.60	7.20	2.35	2.03	65.58	101.18
	90°	2.78	3.45	6.14	6.74	2.21	1.95	59.89	90.86
50 W 6 s	0°	2.59	3.24	6.09	6.51	2.35	2.01	57.99	82.50
	45°	2.67	3.26	6.07	6.55	2.27	2.01	53.34	76.92
	90°	2.66	3.21	5.65	6.15	2.12	1.92	48.82	69.92
25 W 20 s	0°	3.48	4.04	6.60	6.99	1.90	1.73	93.37	121.42
	45°	3.46	3.97	6.77	7.17	1.96	1.81	87.83	113.50
	90°	3.41	3.88	6.34	6.75	1.86	1.74	78.64	101.62

**Table 3:** Lesion sizes computed for different power–duration settings and catheter orientation just after the RF pulse (t_RF_) and at 90 s after the onset of the RF pulse (t_90_) in the thigh model.

		Maximum Depth D (mm)		Maximum Width MW (mm)		Ratio MW/D		Volume (mm^3^)	
Energy Setting	Catheter Orientation	t_RF_	t_90s_	t_RF_	t_90s_	t_RF_	t_90s_	t_RF_	t_90s_
90 W 4 s	0°	2.54	3.47	6.72	7.24	2.65	2.09	73.90	112.61
	45°	2.60	3.48	7.06	7.64	2.72	2.20	71.00	112.40
	90°	2.53	3.36	6.53	7.12	2.58	2.12	62.20	98.69
50 W 6 s	0°	3.13	3.94	6.97	7.50	2.23	1.90	88.78	132.15
	45°	3.15	3.92	6.93	7.51	2.20	1.92	82.74	123.58
	90°	3.11	3.80	6.53	7.12	2.10	1.87	75.73	111.10
25 W 20 s	0°	4.25	5.11	8.03	8.62	1.89	1.69	164.11	225.75
	45°	4.25	5.06	8.11	8.73	1.91	1.73	158.06	218.32
	90°	4.21	4.98	7.76	8.39	1.84	1.68	147.31	203.08

**Table 4:** Percentages of electrical power (P_T_) and delivered energy (E_T_) targeted on the tissue for myocardium (BH) and thigh muscle (TM) model.

		P_T_ (%)		E_T_ (%)	
Energy Setting	Catheter Orientation	BH	TM	BH	TM
90 W 4 s	0°	17.60	25.62	21.31	26.32
	45°	16.91	24.67	19.75	22.61
	90°	16.20	23.88	19.66	21.23
50 W 7.2 s	0°	17.41	25.25	20.69	30.23
	45°	16.77	24.34	19.80	28.78
	90°	16.06	23.52	19.05	27.29
30 W 12 s	0°	17.29	24.98	20.27	30.75
	45°	16.68	24.12	19.40	29.67
	90°	15.96	23.29	18.57	28.81

Power percentages are computed at 0 s (i.e., at the beginning of the RF pulse). Energy percentages are computed at the end of each RF pulse.

## Data Availability

Not applicable.
